# Distribution Dynamics of Recombinant Lactobacillus in the Gastrointestinal Tract of Neonatal Rats

**DOI:** 10.1371/journal.pone.0060007

**Published:** 2013-03-27

**Authors:** Sujin Bao, Libin Zhu, Qiang Zhuang, Lucia Wang, Pin-Xian Xu, Keiji Itoh, Ian R. Holzman, Jing Lin

**Affiliations:** 1 Saint James School of Medicine, Bonaire, The Netherlands Antilles; 2 Yuying Children’s Hospital, Wenzhou Medical College, Wenzhou, China; 3 The First Affiliated Hospital, Wenzhou Medical College, Wenzhou, China; 4 Icahn School of Medicine at Mount Sinai, New York, New York, United States of America; Marie-Joelle Virolle, University Paris South

## Abstract

One approach to deliver therapeutic agents, especially proteins, to the gastro-intestinal (GI) tract is to use commensal bacteria as a carrier. Genus Lactobacillus is an attractive candidate for use in this approach. However, a system for expressing exogenous proteins at a high level has been lacking in Lactobacillus. Moreover, it will be necessary to introduce the recombinant Lactobacillus into the GI tract, ideally by oral administration. Whether orally administered Lactobacillus can reach and reside in the GI tract has not been explored in neonates. In this study, we have examined these issues in neonatal rats. To achieve a high level of protein expression in Lactobacillus, we tested the impact of three promoters and two backbones on protein expression levels using mRFP1, a red fluorescent protein, as a reporter. We found that a combination of an L-lactate dehydrogenase (ldhL) promoter of *Lactobacillus sakei* with a backbone from pLEM415 yielded the highest level of reporter expression. When this construct was used to transform *Lactobacillus casei*, *Lactobacillus delbrueckii* and *Lactobacillus acidophilus*, high levels of mRFP1 were detected in all these species and colonies of transformed Lactobacillus appeared pink under visible light. To test whether orally administered Lactobacillus can be retained in the GI tract of neonates, we fed the recombinant *Lactobacillus casei* to neonatal rats. We found that about 3% of the bacteria were retained in the GI tract of the rats at 24 h after oral feeding with more recombinant Lactobacillus in the stomach and small intestine than in the cecum and colon. No mortality was observed throughout this study with Lactobacillus. In contrast, all neonatal rats died within 24 hours after fed with transformed *E. coli*. Taken together, our results indicate that Lactobacillus has the potential to be used as a vehicle for the delivery of therapeutic agents to neonates.

## Introduction

Probiotics are living microorganisms that confer a health benefit on the host upon ingestion in adequate amounts [1], [2]. Lactic acid bacteria are a large group of bacteria that produce lactic acid as an end product of fermentation. They are commonly used as probiotics because they have long been used in food production and they are safe for human consumption [3]. While the mechanisms remain unclear, clinical studies have demonstrated that supplementation of lactic acid bacteria as a probiotic can significantly reduce the incidence of neonatal necrotizing enterocolitis (NEC), a devastating disease affecting about 5–7% of infants with a birth weight less than 1500 grams and one of the significant causes of mortality and morbidity in premature infants [4], [5], [6], [7].

Therapeutic designs aiming at promoting neonatal intestinal mucosa maturation and protection have become a strategy for prevention and treatment of NEC. A central question is how to effectively deliver therapeutic agents such as mucosa protective factors, anti-inflammatory cytokines or anti-infective agents to the neonatal gut. In the past two decades, whereas significant advances have been made in using Lactococcus as a vehicle to deliver therapeutic agents to the GI tract, in part because expression of exogenous proteins in Lactococcus is well established [8], [9], use of Lactobacillus in this aspect has lagged behind. In particular, expression of exogenous proteins at a high level in Lactobacillus has long been a challenge. However, Lactobacillus offers some advantages over Lactococcus. For example, Lactobacillus species are commensals of the human gut while Lactococcus species are not [10]. In addition, Lactobacillus species typically have a temperature range of 37–41°C for optimal growth, close to the normal human body temperature, compared with 25–30°C for Lactococcus [11]. Further, for certain popular diary products such as yogurt, Lactobacillus but not Lactococcus has been commonly used as a starter culture. As a result, we aimed at developing a Lactobacillus-based system to deliver therapeutic agents to the gastrointestinal tract of neonates. In this work, we used mRFP1, a red fluorescent protein, as a reporter to examine the impact of three different promoters and two different backbones on the expression level of mRFP1. In addition, we used mRFP1 as a marker to label Lactobacillus and examined the retention rate of the recombinant Lactobacillus in the GI tract of neonatal rats.

## Materials and Methods

### Bacteria

The following Lactobacillus strains were used for this work: *Lactobacillus casei subsp. casei* (ATCC 27139^™^), *Lactobacillus acidophilus* (ATCC 11976^™^) and *Lactobacillus delbrueckii subsp. bulgaricus* (ATCC 11842^™^) (Table 1). All these strains were purchased from ATCC (Manassas, Virginia). The *Escherichia coli* DH5α strain was purchased from Life Technologies (Grand Island, New York). Lactobacillus was cultured at 37°C in de Man-Rogosa-Sharpe (MRS) medium (BD-Difco, New Jersey). *E. coli* was cultured in the Circlegrow medium (MP Biomedicals, California). Transformation of *E. coli* followed a standard procedure [12]. Transformed *E. coli* was selected in the presence of 100 µg/ml ampicillin.

**Table 1:** Summary of bacterial strains and plasmids used in this work.

Strains/plasmids	Features	References or sources
**Bacterial strains**		
*Lactobacillus casei subsp. Casei*	Transformation host	ATCC 27139^™^
*Lactobacillus acidophilus*	Transformation host	ATCC 11976^ ™^
*Lactobacillus delbrueckii subsp*. *Bulgaricus*	Transformation host	ATCC 11842^ ™^
*Escherichia coli DH5α*	Transformation host	Life Technologies
**Plasmids**		
pRV85	Emr, containing the dhlL promoter	Ref. [13]
pRSET_B_-mRFP1	Ampr, containing the FLAG-mRFP1 sequence	Ref. [16]
pLEM415	Emr, Ampr; E. coli-Lactobacillus shuttle vector	Ref. [15]
pUCYIT365N	Emr; E. coli-Lactobacillus shuttle vector; containing the emr promoter	GenBank accession No. AB119527
pUCYIT-T7	Emr, derivative of pUCYIT365N	Ref. [17]
pLEM415-ldhL-mRFP1	Emr, Ampr; pLEM415 derivative; containing ldhL-FLAG-mRFP1	This study
pLEM415-emr-mRFP1	Emr, Ampr; pLEM415 derivative; containing emr-FLAG-mRFP1	This study
pLEM415-P59-mRFP1	Emr, Ampr; pLEM415 derivative; containing P59-FLAG-mRFP1	This study
pUCYIT-S1	Emr; derivative of pUCYIT-T7; containing the emr promoter	This study
pUCYIT-ldhL-mRFP1	Emr; pUCYIT-S1 derivative; containing ldhL-FLAG-mRFP1	This study
pUCYIT-emr-mRFP1	Emr; pUCYIT-S1 derivative containing FLAG-mRFP1 downstream of the emr gene	This study
pUCYIT-P59-mRFP1	Emr; pUCYIT-S1 derivative; containing P59-FLAG-mRFP1	This study

Emr, erythromycin resistant; Ampr, ampicillin resistant.

### Transformation of Lactobacillus

Preparation of Lactobacillus competent cells followed a standard protocol provided by Jean-Marc Chatel (MICALIS, INRA, Domaine de Vilvert, Jouy en Josas cedex, France) with some modifications. Specifically, 1 ml of an overnight culture of each Lactobacillus strain was diluted into 100 ml of fresh pre-warmed MRS broth. The culture was incubated at 37°C till the optical density (A_600_) reached 0.6–0.8. The culture was harvested by centrifugation. The cells were washed twice in 20 ml of ice-cold 1× SMEB (0.286 M sucrose, 1 mM MgCl_2_) before being resuspended in 1 ml of 1×SMEB. For each transformation, 0.5 µg of DNA was mixed with 200 µl of 100× concentrated bacteria. The bacterial suspension was electroporated using a Gene Pulser electroporator (Bio-Rad, California) in a cuvette with a 2-mm gap at 2.5 kV, 25 µFD and 200 Ω. Immediately after electroporation, 1 ml of MRS medium was added to the suspension. After incubation at 37°C overnight, serial dilutions were plated on MRS plates containing 25 µg/ml of erythromycin and incubated at 37°C for 2 to 3 days in anaerobic jars.

### Construction of plasmids

**Figure 1:**
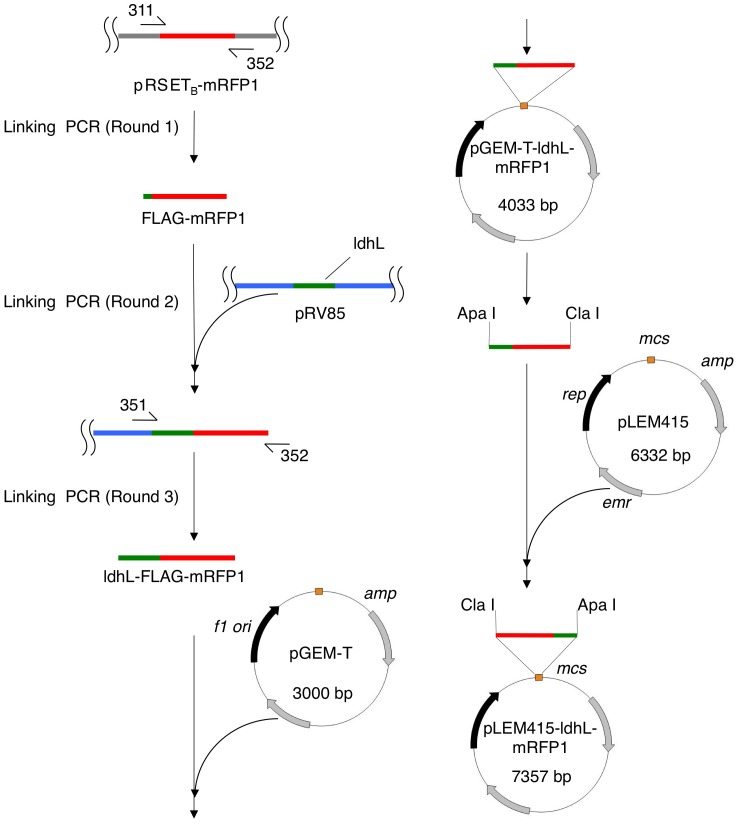
Construction of Lactobacillus-based expression vectors. To generate pLEM415-ldhL-mRFP1, the ldhL promoter and FLAG-mRFP1 were joined by using a 3-round linking PCR technique. The resulting ldhL-FLAG-mRFP1 fragment was then cloned into pGEM-T. Finally, the fragment was released from pGEM-T and cloned into pLEM415. Other expression vectors described in this work were generated in a similar manner. Refer to Materials and Methods for details.

In this work, combinations of three promoters and two backbones were tested. Three promoters used: L-lactate dehydrogenase (ldhL) promoter of *Lactobacillus sakei* derived from pRV85 [13], erythromycin resistance gene promoter (emr) derived from pUCYIT365N (GenBank accession No. AB119527) and P59 promoter [14]. The two backbones used were derived from pLEM415 [15] and pUCYIT365N, respectively. All plasmids used in this work are summarized in Table 1. pLEM415-ldhL-mRFP1 and the other two pLEM415-derived plasmids were generated following a procedure illustrated in Fig.1. Briefly, ldhL-FLAG-mRFP1 that contains the ldhL promoter and FLAG-mRFP1 was amplified by using a 3-round linking PCR technique (Fig. 1): a) In the first round of PCR, FLAG-mRFP1 containing FLAG tag and mRFP1 coding sequences was amplified from pRSET_B_-mRFP1 [16] using two primers 311-ldhl-mRFP and 352-mRFP-NBC (Table 2); b) in the second round of PCR, the ldhL promoter sequence from pRV85 (gift of Monique Zagorec) was joined with FLAG-mRFP1 by allowing pRV85 and the product from the first round of PCR to anneal and extend; c) in the third round of PCR, the ldhL-FLAG-mRFP1 fragment was amplified from the second round of PCR product using two primers 351-ldhl-AXN and 352-mRFP-NBC (Table 2). The product from the final round of linking PCR was cloned into pGEM-T (Promega, WI), giving rise to pGEM-T-ldhL -mRFP1. After verification by DNA sequencing, the ldhL-mRFP1 fragment was released from pGEM-T-ldhL-mRFP1 and inserted into pLEM415 (gift of Pascale Serror) between the same sites, leading to pLEM415-ldhL-mRFP1 (Fig. 1). The emr promoter was amplified from pUCYIT-T7 [17], a derivative of pUCYIT365N, and joined with FLAG-mRFP1 by linking PCR using three primers: 353-emr-AXN, 355-emr-mRFP and 352-mRFP-NBC (Table 2). The P59 promoter sequence was amplified using three overlapping primers: 275-P59-a, 277-P59-b and 276-P59-c (Table 2). The P59 promoter was joined with mRFP1 by linking PCR using three primers: 357-P59-AXN, 278-P59-mRFP and 352-mRFP-NBC (Table 2). emr-FLAG-mRFP1 and P59-FLAG-mRFP1 were cloned into pLEM415 in a manner similar to ldhL-FLAG-mRFP1.

**Table 2:** Primers used in this work.

Primers	Sequences	Application
**To amplify ldhL-FLAG-mRFP1:**		
351-ldhl-AXN	TTAGGGCCCCTCGAGGCTAGCACTGAGAAGTTGCTCTCCCCA	forward, linking PCR
311-ldhl-mRFP	TCCCCGGGTACCGGTAGAAAAAATGACTGGTGGACAGCAAA	forward, linking PCR
352-mRFP-NBC	TTAATCGATAGATCTGCTAGCTTAGGCGCCGGTGGAGTGGCGGCCCT	reverse, linking PCR
**To amplify emr-FLAG-mRFP1:**		
353-emr-AXN	TTAGGGCCCCTCGAGGCTAGCAGTGCAGTATCTTAAAATTTTGT	forward, linking PCR
355-emr-mRFP	GTAATTAAGAAGGAGTGATTACATGACTGGTGGACAGCAAA	forward, linking PCR
352-mRFP-NBC	TTAATCGATAGATCTGCTAGCTTAGGCGCCGGTGGAGTGGCGGCCCT	reverse, linking PCR
**To amplify the P59 promoter:**		
275-P59-a	GGTTTCTTCGCGCATTATAGTCCAAATTCTCTGTTATTCTTTTTTTCTAACTTTTTTCAC	forward, PCR
277-P59-b	TCTAACTTTTTTCACTGTAATTTAAGAACTGTCCCTCTCTATCCAAACTATCTTATATTA	forward, PCR
276-P59-c	ATAATATAAGATAGTTTGGATAATATAAGATAGTT	reverse, PCR
**To amplify P59-FLAG-mRFP1:**		
357-P59-AXN	TTAGGGCCCCTCGAGGCTAGCGGTTTCTTCGCGCATTATAGTCCA	forward, linking PCR
278-P59-mRFP	ACCCATTTGCTGTCCACCAGTCATGTAATCACCTCCTATAATATAAGATAGTTT	reverse, linking PCR
352-mRFP-NBC	TTAATCGATAGATCTGCTAGCTTAGGCGCCGGTGGAGTGGCGGCCCT	reverse, linking PCR

Restriction sites introduced in primers are underlined.

To generate pUCYIT derived plasmids, the T7 polymerase sequence in pUCYIT-T7 was replaced by a short sequence in between XhoI and NheI sites: CTGTGAGAA, giving rise to pUCYIT-S1. To generate pUCYIT-ldhL-mRFP1, ldhL-FLAG-mRFP1 was released from pGEM-T-ldhL-mRFP1 using NheI and cloned into pUCYIT-S1 at the same site. To generate pUCYIT-emr-mRFP1, FLAG-mRFP1 was released from pGEM-T-mRFP1 (Bao, S., unpublished) using XbaI and cloned into pUCYIT-S1 at the NheI site. To generate pUCYIT-P59-mRFP1, P59-FLAG-mRFP1 was released from pGEM-T-P59-mRFP1 using NheI and inserted into pUCYIT-S1 at the same site.

### Determination of Lactobacillus distribution in the GI tract

Pregnant Sprague-Dawley rats were purchased from Taconic Laboratory, New York. Rats were housed in conventional solid-bottom polycarbonate cages (nominal floor area, 930 cm^2^) with standard stainless steel lids and hardwood chip bedding. Cages were changed once a week. Pelleted rat chow was provided ad libitum, and tap water was provided in a water bottle with a sipper tube. The environmental conditions in the animal room were as follows: temperature, 22 to 26 °C; relative humidity, 30% to 60%; lighting, 200 lm/m^2^ at cage level; lights on, 07:00 to 19:00. The female rats were allowed to deliver naturally. On postnatal day 1, a total of 38 pups were divided into three groups. In Group 1 (control group), 12 pups received orally a single dose of saline. In Group 2 (first experimental group), 20 pups received bacterial suspension containing about 6.5×10^8^ CFU of recombinant Lactobacillus transformed with pLEM-415-ldhL-mRFP1. In Group 3 (second experimental group), 6 pups received a single dose of bacterial suspension containing about 6.3×10^8^ CFU of *E. coli* DH5α transformed with pGEM-T-ldhL-mRFP1. At 24, 48, 72 and 96 h after oral administration, three pups from Group 1 and five from Group 2 were sacrificed. The stomach, small intestine, cecum and colon were dissected. Whole segments together with luminal contents were homogenized in phosphate-buffered saline (PBS), serially diluted and plated on MRS plates containing 25 µg/ml of erythromycin. Colonies were counted manually. All pups from Group 3 died at 24 h after oral feeding and no further experiments were performed. All procedures were carried out in accordance with the institutional guideline for the care and use of laboratory animals at the Icahn School of Medicine at Mount Sinai, New York.

### Detection of mRFP1

Images of Lactobacillus colonies on MRS plates were recorded using a Nikon SMZ 1500 dissecting microscope equipped with Nikon LV-TV camera (Nikon, Inc., New York). For fluorescence detection, the pellet of mRFP1 expressing cells was washed once and resuspended in PBS. Cells were fixed in 4% of paraformaldehyde. After washing once with PBS, fixed cells were mounted on a slide and examined using an Axioplan 2 epifluorescence microscope (Carl Zeiss, Inc., New York). Fluorescence images were recorded using an Axiocam digital camera (Carl Zeiss, Inc., New York).

For detection by the western blot, cells were collected from 24 h cultures inoculated using a single colony. Cell pellets were washed once and resuspended in 250 µl of STE solution (6.7% sucrose, 50 mM Tris and 1 mM EDTA). Cells were lysed by adding 250 µl of a lysis solution (200 mM NaOH and 1% SDS) in the presence of protease inhibitors for 5 minutes. Lysates were cleared by centrifugation. The total protein concentration was determined by using a BCA assay (Thermo Scientific, New York). An equal amount of protein in a total of 0.9 µg from each sample was loaded onto an 8% SDS-PAGE gel. Proteins were transferred onto a polyvinylidene difluoride (PVDF) membrane (Roche, Indiana) using a Bio-Rad electrotransfer system (Bio-Rad, California). The FLAG-mRFP1 protein was detected using an M2 anti-FLAG antibody (Sigma-Aldrich, Missouri). An anti-mouse IgG-HRP conjugate (Cell Signaling, Massachusetts) was used as the secondary antibody. Chemical luminescence signals were captured using the Fujifilm LAS-3000 imaging system equipped with a digital camera (Fuji Medical Systems USA, Connecticut). Intensities of proteins bands were quantified using ImageJ [18].

## Results

### Expression of mRFP1 at a high level in *Lactobacillus casei*

**Figure 2:**
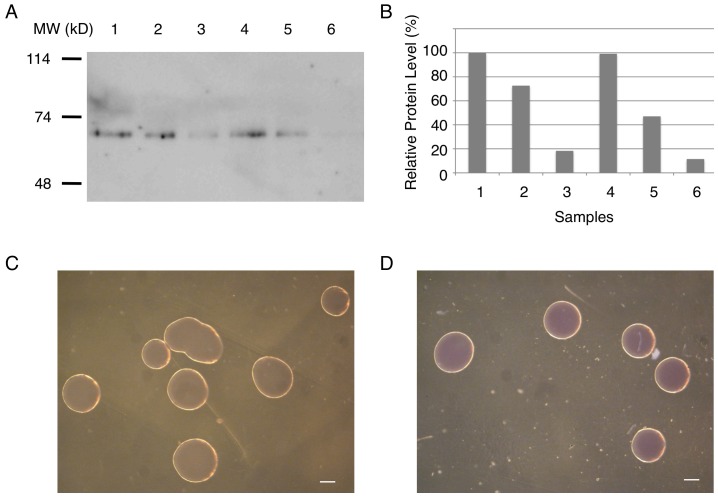
Different expression levels of Lactobacillus-based expression vectors. A) Different levels of mRFP1 expression in *Lactobacillus casei* were detected by Western blot. A total of 0.9 µg of the whole lysate was loaded into each lane. Lane 1, pLEM415-ldhL-mRFP1; lane 2, pLEM415-emr-mRFP1; lane 3, pLEM415-P59-mRFP1; lane 4, pUCYIT-ldhL-mRFP1; lane 5, pUCYIT-emr-mRFP1; lane 6, pUCYIT-P59-mRFP1. B) Quantification of band intensities from Western blot in Panel A. The expression level of pLEM415-ldhL-mRFP1 was set to 100%. Sample numbers correspond to lanes shown in Panel A. C) Wild type *Lactobacillus casei* colonies under visible light. D) Colonies of *Lactobacillus casei* transformed with pLEM415-ldhL-mRFP1 appeared pink under visible light. Scale bars, 100 µm.

A green fluorescent protein (GFP) and its variants have been broadly used as a reporter or marker. However, a high rate of fluorescence quenching has been observed in GFP at acidic pH [19]. Since Lactobacillus cultures produce lactic acid, detection of GFP in Lactobacillus in some cases has been problematic [20]. To circumvent this problem, in this study we used mRFP1, a red fluorescent protein, known to tolerate a low pH [16]. To achieve a high level of protein expression in Lactobacillus, we tested the impact of three promoters and two backbones on protein expression since choices of the promoter and the backbone often have a significant impact on the expression level of a target gene. Each construct was electroporated into *Lactobacillus casei*. A high level of expression was observed with the ldhL promoter. The highest level of mRFP1 expression was found with pLEM415-ldhL-mRFP1, a construct that combines the pLEM415 backbone and the ldhL promoter (Fig. 2A–B). The mRFP1 expression was so high that bacterial colonies appeared pink under visible light (Fig. 2C–D). In contrast, a lower level of mRFP1 expression was observed with the emr promoter. The amount of the mRFP1 protein expressed by pLEM-emr-mRFP1 and pUCYIT-emr-mRFP1 was about 73% and 47%, respectively, of that by pLEM415-ldhL-mRFP1 (Fig. 2A–B). A substantially lower level of expression was observed with the P59 promoter (Fig. 2A–B): levels of mRFP1 expressed by pLEM415-P59-mRFP1 and pUCYIT-P59-mRFP1 were only 18% and 11%, respectively, of that by pLEM415-ldhL-mRFP1. The combination of the pUCYIT365N backbone and the P59 promoter led to the lowest level of expression.

**Figure 3:**
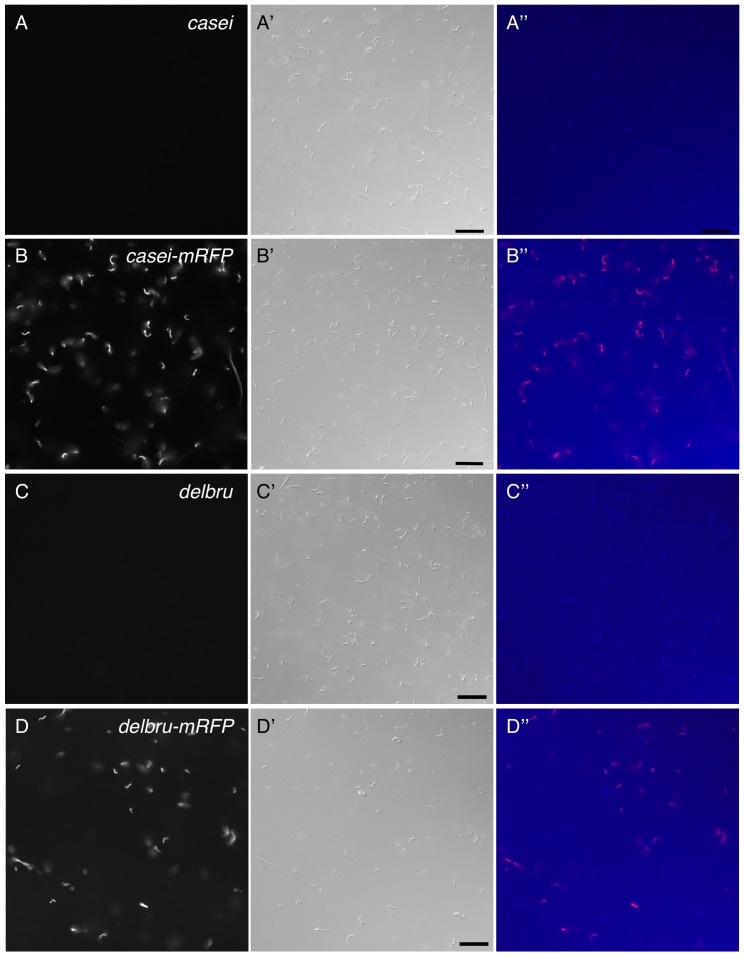
mRFP1 can be expressed broadly in Lactobacillus. The pLEM415-ldhL-mRFP1 vector was transformed into *L. casei* (B) and *L. delbrueckii* (D). mRFP1 was detected in both strains. Wild type strains were used as control (A,C). mRFP1 expression (red) is shown in the left panels and DIC images (purple) in the middle. Merged views are shown in the right panels. Scale bars, 20 µm.

Due to its ability to express an exogenous protein at a high level in *Lactobacillus casei*, pLEM415-ldhL-mRFP1 was used to label *Lactobacillus casei* for further analyses. In fact, the high level of mRFP1 expression was not limited to *Lactobacillus casei*. When pLEM415-ldhl-mRFP1 was transformed into *Lactobacillus delbruekii* and *Lactobacillus acidophilus*, an equivalent level of mRFP1 was also observed (Fig. 3A–D and data not shown) and the colonies appeared pink under visible light, indicating pLEM415-ldhL-mRFP1 can be broadly utilized to label Lactobacillus.

### Dynamic localization of *Lactobacillus casei* in the GI tract of neonatal rats

**Figure 4:**
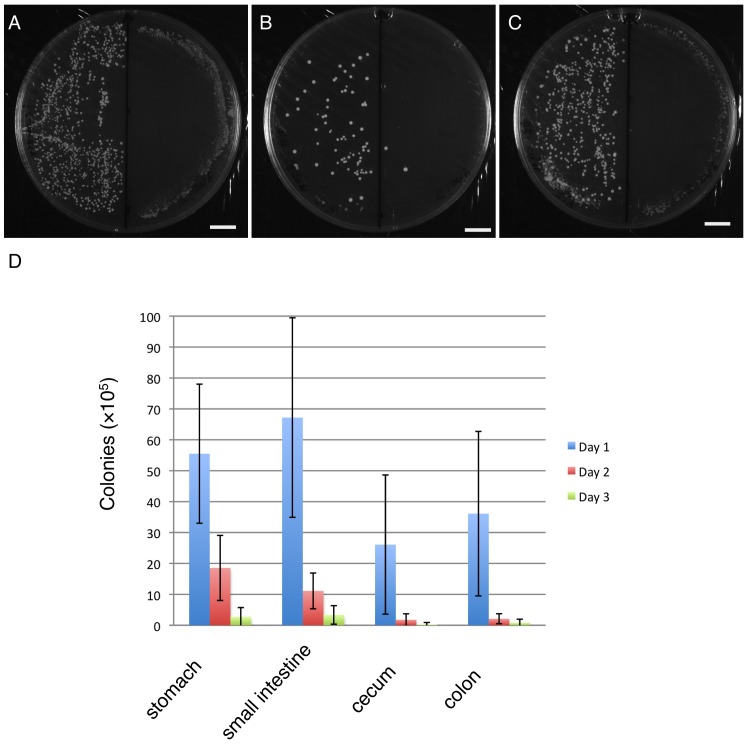
Dynamic distribution of Lactobacillus in the GI tract of neonatal rats. Neonatal rats were fed with *L. casei* expressing mRFP1. Animals were sacrificed on Days 1, 2, 3 and 4 after gavage. The gastrointestinal homogenates were diluted and plated on MRS plate in the presence of erythromycin. Control rats were fed with saline only. Homogenates from experimental groups were plated on the left half of each plate and those from controls on the right half (A–C). Colonies were found in animals fed with labeled bacteria on Day 1. A) Stomach; B) cecum; C) colon. Scale bars, 10 mm. D) Entire colony counts. Samples were collected on Days 1 (blue), 2 (red) and 3 (green). Note: standard deviation was used to measure variability.

To test whether *Lactobacillus casei* can be retained in the GI tract of neonates, we introduced 6.5×10^8^ CFU of mRFP1-labeled *Lactobacillus casei* into neonatal rats on post-natal day 1 by gavage. At 24 h after oral administration of a single dose of Lactobacillus, about 3% of bacteria (1.9×10^7^ CFU) were retained in the GI tract without taking into account proliferation of the bacteria in the GI tract. These bacteria were found across all segments of the GI tract (Fig. 4A–C), but bacterial distributions were not even along the GI tract. In fact, a larger number of *Lactobacillus casei* presided in the stomach and small intestine than in the cecum and colon: about 30% (5.6×10^6^ CFU) and 35% of bacteria (6.7×10^6^ CFU) were found in the stomach and small intestine compared with 14% (2.6×10^6^ CFU) and 20% (3.6×10^6^ CFU) in the cecum and colon, respectively (Fig. 4D). This pattern is very reminiscent of natural distribution of Lactobacillus in the human GI tract where Lactobacillus predominates in the small intestine in some elderly individuals [21]. This distribution pattern in the neonatal rats largely remains unchanged till 72 h after oral feeding. At 48 h, the total bacteria retained in the GI tract dropped to 0.5% (3.4 ×10^6^ CFU) (Fig. 4D). At 72 h, only very few bacteria (about 0.1%) were found in the GI tract (Fig. 4D). At 96 h, recombinant *Lactobacillus casei* was almost undetectable using our assay. Although these colonies retained the antibiotic resistance, we observed that only about 31% of colonies preserved fluorescence (n = 352) when a total of 1144 colonies were examined. It is not clear why over 2/3 of colonies retained the antibiotic resistance but lost fluorescence; we are currently investigating this issue. Noticeably, during the course of this study, no mortality was observed in neonatal rats treated with either saline or recombinant Lactobacillus. In contrast, all neonatal rats treated with the *E. coli* DH5α strain transformed with pGEM-T-ldhL-mRFP1 died within 24 h after gavage (n = 6), precluding its use as a delivery vehicle. Taken together, these results indicate that a portion of *Lactobacillus casei* can be retained in the GI tract of neonatal rats for at least 24 hours.

## Discussion

In this study, we achieved a high level of protein expression in Lactobacillus by combining an ldhL promoter from *Lactobacillus sakei* with a backbone from pLEM415. In addition, we examined the expression of mRFP1 in 3 strains of Lactobacillus: *Lactobacillus casei*, *Lactobacillus delbrueckii* and *Lactobacillus acidophilus*. Expression levels of mRFP1 in all these strains were high. Using *Lactobacillus casei* that expressed mRFP1, we showed that a portion of the recombinant bacteria was retained in the GI tract of neonatal rats for at least 24 hours. Our results indicate that the recombinant Lactobacillus has the potential to be used as a vehicle for the delivery of therapeutic agents to the mucosa of GI tract.

Low levels of protein expression in Lactobacillus have hindered the wide application of Lactobacillus in therapeutic design. As a result, in the past decade many efforts have been made to improve the level of protein expression in this genus of Gram-positive bacteria. To date, constitutive expression of GFP in Lactobacillus has been reported [20], [22]. However, GFP protein is not detectable under visible light and therefore its detection requires a fluorescence microscope. In addition, GFP and its variants are often sensitive to acidic pH. Due to quenching of GFP at a low pH [19], detection even with a microscope has been problematic. Although in some instances the GFP protein expressed in Lactobacillus can be visualized after taking additional measures such as neutralizing cells with a neutral buffer [20], the degree of success with these measures has been variable in our hands. To overcome these problems, we have used mRFP1 as a reporter, which is known to be stable at a low pH [16]. To achieve a high level of protein expression in Lactobacillus, we have tested different combinations of two backbones and three promoters. We found for a given backbone the ldhL promoter overall yielded the highest level of reporter expression and the P59 promoter the lowest. The emr promoter yielded an intermediate level of reporter expression. For a given promoter, the pLEM415 backbone conferred a higher level of expression than pUCYIT365N backbone. Consistently, the combination of the ldhL promoter and pLEM415 backbone yielded the highest level and the combination of the P59 promoter and pUCYIT365N backbone led to the lowest level of protein expression. While different expression levels conferred by different promoters with a given backbone clearly indicate differential strengths of the promoters, the reason why the same promoter gave rise to different levels of protein expression when combined with different backbones may not be straightforward. At this stage, it is not clear whether the copy number of pLEM415 was higher than that of pUCYIT365N in a host bacterial strain or whether the secondary structure of plasmid DNA resulting from a particular combination makes the same promoter in pLEM415 more accessible to the transcriptional machinery than in pUCYIT365N. Of note also is the unusual migrating behavior of the FLAG-mRFP1 protein, which has a molecular weight of 28 kDa. In a Western blot, this protein migrated at 65 kDa (Fig. 2A). One possibility is that the stop codon used in these constructs was leaky, leading to a fusion protein that is larger than FLAG-mRFP1. Alternatively, FLAG-mRFP1 formed a dimer under the SDS-PAGE condition. Currently we do not have any further evidence to distinguish these possibilities.

Our results demonstrate that Lactobacillus can be retained in all segments of the gastro-intestinal tract in neonatal rats for at least 24 hours after oral administration. In particular, a larger number of the recombinant bacteria were found in the stomach and small intestine than in the cecum and colon. One possibility is that the stomach and small intestine are preferred segments for Lactobacillus to colonize since Lactobacillus predominates in the small intestine in some individuals [21]. Alternatively, these results may reflect larger luminal surface areas of the stomach and small intestine than those of the cecum and colon. Further studies are needed to clarify this issue.

Notwithstanding this, we have noticed that the retention rate of Lactobacillus is low in the GI tract of neonatal rats. Even without taking into account proliferation of Lactobacillus *in vivo*, only about 3.0% of Lactobacillus was retained in the GI tract at 24 h after oral feeding. From a clinical perspective, a low retention rate may actually be advantageous since complete removal of recombinant Lactobacilli from the GI tract after oral administration within a limited period of time will be essential for eliminating any potential unwanted long-lasting side effect of recombinant Lactobacillus on the host after therapeutic treatments. On the other hand, for therapeutic purposes, a retention rate of 3% may not be large enough to elicit strong immune responses in certain applications. One way to address this issue could be to administer neonates with multiple doses of recombinant Lactobacillus. Since Lactobacillus inhabits the small intestine, cecum and colon in humans [21], [23], [24], an alternative approach is to use Lactobacillus species with a high capacity to colonize the human GI tract. Further studies are needed to isolate those species that are also capable of expressing a target protein at a desired level. Notably, during the course of this study, no mortality was observed in neonatal rats fed with Lactobacillus. In contrast, all neonates administered with the transformed E. coli DH5α strain died within 24 hours after gavage, precluding the use of *E. coli* as a vehicle in neonates. Taken together, our results indicate the Lactobacillus is safe and has the potential to be used as a vehicle to deliver therapeutic agents to the gastro-intestinal tract of neonates.
